# Machine learning-based prediction of COVID-19 mortality using immunological and metabolic biomarkers

**DOI:** 10.1186/s44247-022-00001-0

**Published:** 2023-02-03

**Authors:** Thomas Wetere Tulu, Tsz Kin Wan, Ching Long Chan, Chun Hei Wu, Peter Yat Ming Woo, Cee Zhung Steven Tseng, Asmir Vodencarevic, Cristina Menni, Kei Hang Katie Chan

**Affiliations:** 1 Department of Biomedical Sciences, City University of Hong Kong, Hong Kong SAR, China; 2 Computational Data Science Program, Addis Ababa University, Addis Ababa, Ethiopia; 3 Department of Electrical Engineering, City University of Hong Kong, Hong Kong SAR, China; 4 Department of Neurosurgery, Kwong Wah Hospital, Hong Kong SAR, China; 5 Department of Medicine and Geriatrics, Kwong Wah Hospital, Hong Kong SAR, China; 6 Innovative Medicines, Novartis Pharma GmbH, 90429 Nuremberg, Germany; 7 Department of Twin Research, King’s College London, London, UK; 8 Department of Epidemiology and Center for Global Cardiometabolic Health, School of Public Health, Brown University, Providence, RI USA

**Keywords:** Biomarkers, Machine learning, Random forest classifier, Deep neural network, COVID-19

## Abstract

**COVID-19 mortality prediction**

**Background** COVID-19 has become a major global public health problem, despite prevention and efforts. The daily number of COVID-19 cases rapidly increases, and the time and financial costs associated with testing procedure are burdensome.

**Method** To overcome this, we aim to identify immunological and metabolic biomarkers to predict COVID-19 mortality using a machine learning model. We included inpatients from Hong Kong’s public hospitals between January 1, and September 30, 2020, who were diagnosed with COVID-19 using RT-PCR. We developed three machine learning models to predict the mortality of COVID-19 patients based on data in their electronic medical records. We performed statistical analysis to compare the trained machine learning models which are Deep Neural Networks (DNN), Random Forest Classifier (RF) and Support Vector Machine (SVM) using data from a cohort of 5,059 patients (median age = 46 years; 49.3% male) who had tested positive for COVID-19 based on electronic health records and data from 532,427 patients as controls.

**Result** We identified top 20 immunological and metabolic biomarkers that can accurately predict the risk of mortality from COVID-19 with ROC-AUC of 0.98 (95% CI 0.96-0.98). Of the three models used, our result demonstrate that the random forest (RF) model achieved the most accurate prediction of mortality among COVID-19 patients with age, glomerular filtration, albumin, urea, procalcitonin, c-reactive protein, oxygen, bicarbonate, carbon dioxide, ferritin, glucose, erythrocytes, creatinine, lymphocytes, PH of blood and leukocytes among the most important biomarkers identified. A cohort from Kwong Wah Hospital (131 patients) was used for model validation with ROC-AUC of 0.90 (95% CI 0.84-0.92).

**Conclusion** We recommend physicians closely monitor hematological, coagulation, cardiac, hepatic, renal and inflammatory factors for potential progression to severe conditions among COVID-19 patients. To the best of our knowledge, no previous research has identified important immunological and metabolic biomarkers to the extent demonstrated in our study.

**Supplementary Information:**

The online version contains supplementary material available at 10.1186/s44247-022-00001-0.

## Background

Coronavirus disease 2019 (COVID-19) is an infection caused by severe acute respiratory syndrome coronavirus 2. This virus was first identified in Wuhan, China, in December 2019 and has since led to a global pandemic that has affected more than 254 million people worldwide as of November 16, 2021, according to World Health Organization (WHO).

Following the initial outbreak, COVID-19 rapidly spread to all parts of the world and has since become the most significant global public health crisis of the last 2 years. This pandemic is highly challenging because no specific or fully effective treatment is currently available, and the disease dynamics are not properly understood.

Coronaviruses compromise a large family of viruses that are known to cause illnesses ranging from the common cold to more severe diseases, such as Middle East respiratory syndrome (MERS) and severe acute respiratory syndrome (SARS). According to statistics from the WHO, issued on March 30,2020, the average mortality rate among confirmed COVID-19 cases was 4.6%, with a range from 0.2% to 15% depending on the affected individuals’ age health and immune status and location of residence [1, 2].

COVID-19 is mostly spread through respiratory droplets, produced by coughing or sneezing, and this spread has occurred at an alarmingly rapid pace, moving from one city to whole countries and taking many lives. The rapid global spread of COVID-19 has led to significant effects in roughly 213 countries and territories. Since December 2019, more than 250 million cases of COVID-19 and 5 million related deaths have been registered. Numerous risk variables have been linked to poor outcomes, including lymphocyte counts [3], high levels of various inflammatory or coagulation indicators [4] and serum levels of various cytokines [5].

The clinical subtype of COVID-19 has been identified using a combination of clinical characteristics and biochemical markers like D-Dimer, C-Reactive Protein and lactic dehydrogenase [6]. Machine learning (ML) techniques have been applied to such heterogeneous multimodal data for the classification of COVID-19 patients. For example, ML has been used to diagnose COVID-19 pneumonia, stratify patients, and construct a prediction model of dissemination patterns [7]. Previous studies have identified important risk factors associated with COVID-19 mortality, such as increased age, cardiovascular disease, chronic pulmonary illness, diabetes, hypertension, smoking history, and obesity [8, 9]. Several studies have used machine learning algorithms to predict COVID-19 mortality [10–16]. The accurate prognosis of COVID-19 clinical outcome is more difficult owing to the wide range of illness severity that might be beneficial for appropriate triage, limited resources and enhance patient care within health-care systems.

Our proposed model is expected to greatly benefit COVID-19 prevention, diagnosis and management efforts targeting the general population.

## Methodology

**Fig. 1:**
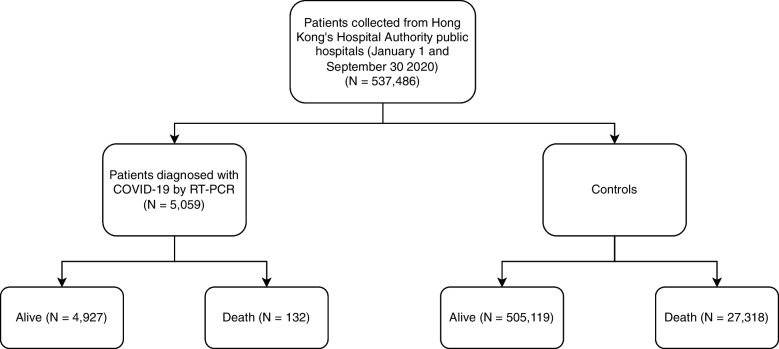
Details of dataset provided by Hong Kong’s Hospital Authority

This study included inpatients from Hong Kong’s Hospital Authority public hospitals between January 1, and September 30, 2020, who were diagnosed with COVID-19 using real RT-PCR tests. The Hospital Authority is Hong Kong’s primary public healthcare institution responsible for delivering hospital-based care for 90% of inpatient bed-days in the city. Data was obtained from the Hospital Authority Data Collaboration Laboratory, a big analytics platform that was established for the purpose of facilitating biotechnological research. Figure 1 shows the details of the original dataset and data size for each class.An additional document file shows more details of dataset features [see Additional file 1].

**Fig. 2:**
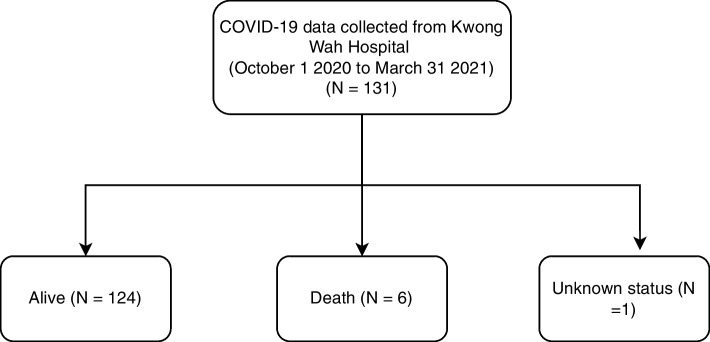
Details of dataset provided by Kwong Wah Hospital

We developed five machine learning models to predict the mortality of COVID-19 patients, using data from their electronic medical records for training. We performed statistical analysis to compare the trained machine learning models using data from a cohort of 5,059 patients (median age = 46 years, 95% confidence interval (CI): [45,46.1]; 49.3% male) who had tested positive for COVID-19 based on electronic records and data from 532,427 patients as controls. Figure 2 shows dataset details from an independent cohort from a public hospital - Kwong Wah Hospital (131 patients), which was used for model validation. At the data cleaning stage, we removed unnecessary (e.g., Patient personal identifiers), redundant data elements and unlabeled data samples.

ML models were built using Python with IDE provided by PyCharm 2021.2.2 (Runtime version: 11.0.12+7-b1504.28 amd64) with OpenJDK 64-Bit Server VM by JetBrains s.r.o. JDK version, Anaconda3 and Anaconda Navigator 2.0.4 was the project interpreter. The ML models were built based on Keras with version 2.7.0 and Tensorflow with version 2.6.1.

### Data preprocessing

In the original dataset, there were 20 data tables including different types of data, for example: 435 types of different laboratory result, immunization injection, smoke status, alcohol status, family history, weight, and height. First, the outcome table which include the COVID-19 information was the main table and it was merged with other tables with the mapping key (project-specific serial number for each patient). An additional document file shows more details of data preprocessing for data tables [see Additional file 2].

### Feature selection, data splitting and imputation

**Fig. 3:**
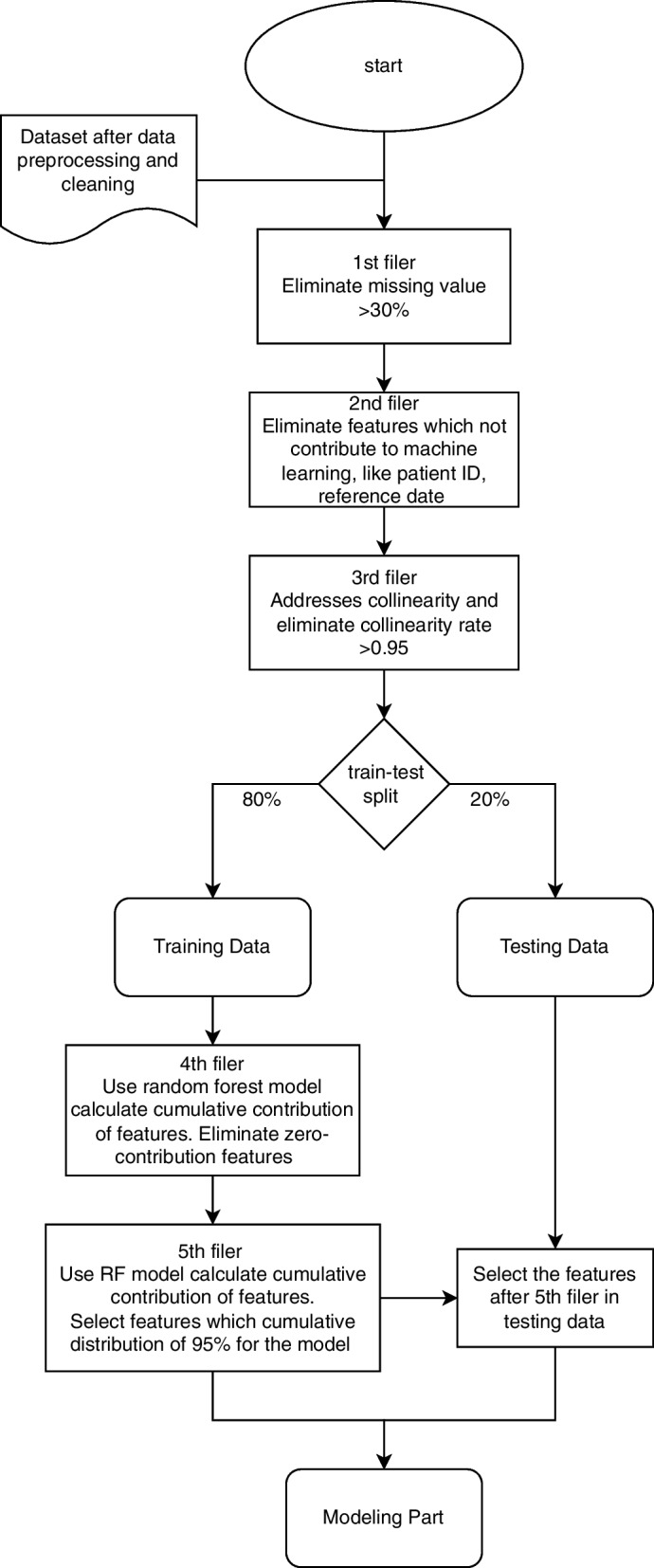
Flow Chart for variable selection

A total of 171 features were extracted from the original dataset, including 63 immunological and metabolic biomarkers. We consulted with a clinical team to ensure that all relevant features were extracted. The top 20 features were selected using different filter and wrapper methods to identify the most informative biomarkers. Figure 3 shows the flowchart of feature selection, five layers of feature selection were applied in this study. For the first filter, the overall threshold for missing values was set as 30%; therefore, a feature was eliminated if more than 30% of relevant values were missing. The second filter was set to eliminate features that did not contribute significantly to machine learning, such as patient identification numbers and the reference dates of different features. The third feature selection feature addressed collinearity. It was included to avoid feature duplication, which may have inappropriately placed higher importance on similar types of information in the model. Two laboratory tests were shown to exhibit high collinearity and to contain 95% similar information in the data set.

After the third filter, the data splitting and imputation were implemented before the fourth filter for avoiding data leakage problem. The train-test ratio for the RF and SVM models is 80:20 and data was split randomly. The DNN model split the training set into the training set and validation set by 80:20 ratio. An iterative imputation method MissForest was applied to replace the missing values in the training set.

The fourth filter applied a random forest model to training data to determine the importance of the features. Sixty-eight features were identified to have zero importance after one hot-encoding.

The fifth filter simplified the machine learning model to achieve high efficiency and reduce the running time for model training. This filter selected 53 features required for cumulative importance of 95% after one hot encoding; additionally,119 features were not found to contribute to cumulative importance of 95%.

The selected biomarkers, (i.e., features) were categorized into six groups: hematological, inflammatory, coagulation, hepatic, muscle and renal. An additional document file shows more details of selected biomarkers [see Additional file 3].

After the feature selection processing, this study applied SMOTE in the training set and validation set for the minority group oversampling to handle the imbalanced data size of each class.

### Machine learning algorithms and performance metrics

This study applied several machine learning algorithms such as DNN, RF, SVM models with linear, polynomial, radial basis function and sigmoid kernels.

Standard evaluation metrics, such as accuracy, sensitivity, precision and specificity, were used to quantify the performance of the predictive models.

A receiver operating characteristic curve analysis was conducted to explore the balance between the true-positive (sensitivity) and false-positive rates (specificity) for each model. The classifiers were compared using the area under the curve (AUC).

Accuracy, sensitivity, and specificity are defined as follows:

$$\begin{aligned} Accuracy=\frac{TP+TN}{TP+FN+TN+FP} \end{aligned}$$

Where the model accuracy represents the proportion of test records that are correctly classified.

$$\begin{aligned} Sensitivity=\frac{TP}{TP+FN} \end{aligned}$$

where TP, TN, FP and FN represent the numbers of true positives, true negatives, false positives and false negatives, respectively.

The threshold of outlier is defined as greater than or less than 3 standard deviations from the mean. Outliers were removed and replaced by mean for numerical features and mode for the categorical features, and data were scaled to a range of [-1, 1] using a standardization formula and min-max normalization. Standardization and normalization prevent domination of the model by features with greater numeric values. The following standardization formula was used:

$$\begin{aligned} Z=\frac{X-\overline{X}}{\sigma (X)/\sqrt{n}} \end{aligned}$$

Min-max normalization was calculated using the following formula:

$$\begin{aligned} Z=minRange+ \frac{(maxRange - minRange) * (unscaledData - min)}{(max - min)} \end{aligned}$$

**Fig. 4:**
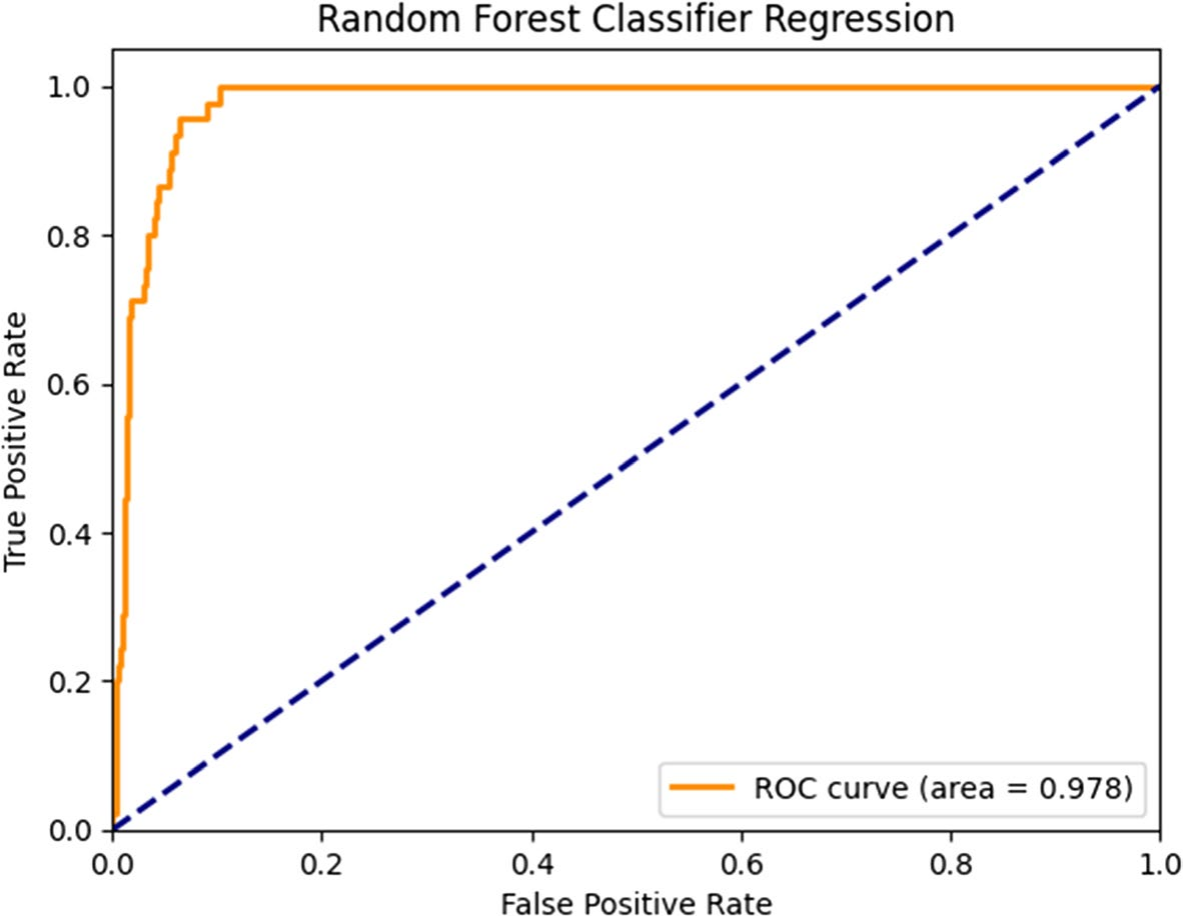
Performance of the best model (RF)

The random forest algorithm was determined to achieve the best performance and accuracy, as indicated in Fig. 4.

The performance of each model was evaluated at different stages using training sets of 1,000, 2,000, 3,000 and 4,000 patient records to determine how the number of records influenced the model performance.

### Statistical description of the data

In this study, the data frame of the various features of COVID-19 patients was first split into two data frames of features according to the patients’ survival status (survivors and deceased), which are hereafter referred to as the surviving group and deceased group respectively. The following statistical analysis were applied:

1. *Outliers:* Outliers were detected and removed from each table and continuous feature using Turkey’s method. Briefly, after removing the missing values, the interquartile range (IQR), upper quantile (Q3) and lower quantile (Q1) were computed for each future. According to Tukey’s method, an outlier is defined as a value that is higher than Q1 by 1.5 times of IQR. These outliers were removed and replaced as NA.
2. *Report of the robust descriptive statistics:* After removing the outliers, the median, Q3 and Q1 are computed for the remaining data in both the surviving and deceased group tables and for each future. The results are shown in Table 1.
3. *Hypothesis tests comparing features between Survivors and deceased patients:* For each feature, data were compared between the surviving and deceased groups via two-tailed hypothesis testing. The null hypothesis was formulated as:
  $$\begin{aligned} H_0:\upsilon _{survivors} =\upsilon _{deceased} \end{aligned}$$
  The Wilcoxon rank sum test was performed for every feature with *p*-value computed using ‘wilcox.test’ method on the R platform. The *p*-values are reported in the Table 1.

**Table 1:** Descriptive statistical characteristics of the included feature

Features: Median (IQR)	Alive Group (*N*=4,887)	Dead Group (*N*=132)	*P*-value
**Demographic factors**			
Age (years)	43(28 - 59)	83(76 - 88)	< 0.001
Height(m)	1.61(1.54 - 1.68)	1.57(1.51 - 1.60)	0.012
BMI(kg/M^2^)	25.47(22.9 - 28.04)	24.02(22.4 - 26.3)	0.119
Weight(kg)	65.7(58.2 - 75.3)	62.8(54.4 - 70.4)	0.275
**Liver function related tests:**			
Alanine aminotransferase in serum or plasma ( U/L)	21 (15 - 30)	17 (12 - 25)	< 0.001
Alkaline phosphatase in serum or plasma ( U/L)	65 (54 - 78)	77 (62 - 97)	< 0.001
Aspartate aminotransferase in serum or plasma (U/L)	25 (21 - 34)	28 (23 - 52)	0.004
Gamma glutamyl transferase in serum or plasma (U/L)	32 (21 - 56)	46.9 (34.8 - 108.3)	0.002
Direct bilirubin in serum or plasma – umol/L	9.6 (6.2 - 12.0)	29.0 (18.7 - 46.9)	< 0.001
Bilirubin in serum or plasma (umol/L)	7.6 (5.6 - 10.3)	8.0 (6.0 - 11.5)	0.214
**Liver function related tests:**			
Albumin in urine (mg/L)	8.35 (2.99 - 20.86)	11.31(4.44 - 16.00)	0.844
**Inflammatory biomarkers:**			
C reactive protein in serum or plasma (mg/L)	2.9 (1.0 - 6.4)	25.8 (6.3 - 69.5)	< 0.001
Erythrocyte sedimentation rate (mm/hr)	21 (11 - 36)	56.5 (33.75 - 81.75)	< 0.001
**Pancreas function related tests:**			
Amylase in serum or plasma (U/L)	64 (51 - 81)	74 (58 - 101)	0.003
**Hematological biomarkers:**			
Base excess in blood (mmol/L)	-0.2 (-1.8 - 1.6)	-1.4 (-4.2 - 1.3)	0.002
Bicarbonate in blood (mmol/L)	24.0 (21.4 - 26.3)	23.0 (19.1 - 25.1)	0.001
Bicarbonate in serum – mmol/L	24.39 (21.87 - 26.23)	21.75 (20.04- 23.68)	0.005
Calcium corrected for albumin in serum or plasma (mmol/L)	2.28 (2.21 - 2.34)	2.31 (2.22 - 2.38)	0.006
Calcium in serum or plasma (mmol/L)	2.28 (2.21 - 2.36)	2.21 (2.11 - 2.33)	< 0.001
Calcium.ionized in blood ( mmol/L)	1.11 (1.04 - 1.15)	1.10 (1.05 - 1.14)	0.816
Carboxyhemoglobin/Hemoglobin.total in blood (%)	0.5 (0.3 - 0.9)	0.3 (0.3 - 0.3)	0.044
Deoxyhemoglobin/Hemoglobin.total in blood (%)	3.5 (2.5 - 4.2)	3.7 (2.2 - 29.6)	0.620
Chloride in Serum or Plasma (mmol/L)	102.0 (100.0 - 104.0)	102.9 (100.8 - 106.2)	0.020
Carbon dioxide [Moles/volume] in Blood (mmol/L)	26.0 (23.0 - 28.5)	25.7 (20.4 - 27.0)	0.134
Carbon dioxide [Partial pressure] in blood (kPa)	5.0 (4.3 - 5.8)	4.7 (4.1 - 5.5)	0.063
**Carcinogenic biomarkers:**			
Carcinoembryonic Ag in serum or plasma (ng/mL)	2.40 (1.46 - 3.70)	3.55 (2.75 - 5.30)	0.004
Alpha-1-Fetoprotein in serum or plasma( ng/mL)	2.19 (1.65 - 2.89)	1.61 (1.33 - 1.86)	0.087
**Cardiac function related tests:**			
Cholesterol.in LDL in Serum or Plasma by Calculated – mmol/L	2.41 (1.89 - 3.10)	1.88 (1.40 - 2.20)	< 0.001
Cholesterol.in HDL in serum or plasma (mmol/L)	1.2 (1.0 - 1.5)	1.2 (1.0 - 1.4)	0.543
Cholesterol non HDL in serum or plasma (mmol/L)	3.10 (2.50 - 3.83)	2.43 (1.98 - 3.13)	< 0.001
Cholesterol.total/Cholesterol in HDL in serum or plasma (mol/mol)	3.57 (2.91 - 4.48)	3.23 (2.57 - 3.85)	0.002
Cholesterol in Serum or Plasma – mmol/L	4.4 (3.7 - 5.2)	3.7 (3.3 - 4.1)	< 0.001
Troponin T.cardiac in serum or plasma (ng/L)	5.0 (3.0 - 7.1)	21.1 (8.7 - 34.9)	<0.001
Creatine kinase in serum or plasma (U/L)	81.0 (58.8 - 117.0)	90.5 (58.0 - 175.5)	0.033

#### Feature importance and accuracy with different data size

From Table 1, several aspects of factors were compared between the alive and dead groups using Wilcoxon’s rank-sum tests. The null hypotheses were set to be that the groups had insignificantly different mean values among the listed risk factors. The *p*-values of the tests were obtained and used to select the candidates for the subsequent *p*-values corrections due to the multiple hypothesis tests. Using Bonferroni correction, we can minimize the high Type I error rate raised by multiple hypothesis tests and conclude the comparisons. At first, 22 risk factors were selected as candidates for subsequent Bonferroni correction as their corresponding *p*-values, obtained from the Wilcoxon’s rank-sum tests, were smaller than 0.05, the significance level. Then, Bonferroni correction was implemented on these *p*-values from those 22 risk factors by simply multiplying the *p*-values with the number of hypothesis tests. The corrected *p*-values and their corresponding factors were selected according to the significance level of 0.05. Those factors with corrected *p*-values smaller than 0.05 were selected as the factors that show the significant difference between alive and dead groups. It turned out that 14 factors showed a significant difference between the alive and dead groups after the multiple comparisons adjustment. They are ages in the demographic factors;alanine aminotransferase, aspartate aminotransferase, gamma-glutamyl transferase and direct bilirubin in serum or plasma among the liver function-related tests; C-reactive protein and erythrocyte sedimentation rate which belong to inflammatory biomarkers; some hematological biomarkers including base excess in blood, bicarbonate in blood and calcium in serum or plasma; and also several cardiac function related biomarkers including cholesterol in LDL, cholesterol of non HDL, cholesterol total/ cholesterol in HDL and cholesterol in serum or plasma and also troponin T. cardiac in serum or plasma. These are the risk factors that statistically significantly different between the alive and dead groups of COVID-19 patients.

### Model

#### Deep neural network

The DNN model used grid search hyperparameter tuning tools to adjust the number of neurons and layers. The DNN model applied one input layer, two fully connected hidden layers, and one output layer. The first hidden layer had 52 neurons, and the second hidden layer had 13 neurons. RandomNormal was chosen as the initializer to initialize the random normal values, Relu and Adadelta were used as the optimizer in the hidden layers and output layer. EarlyStopping was applied to optimize the number of epochs to avoid the over-fitting problem, and validation loss is the indicator for the EarlyStopping monitoring.

#### Random forest classifier

The RF model applied the ‘Gini’ impurity metric (mean decrease in impurity) to determine the feature importance. For the design of RF model, GridSearchCV was used to adjust the value of parameters. After the hyper-parameters tuning, the maximum depth is set at 8, the maximum features set at “sqrt”, the minimum samples leaf set at 1, the minimum samples split is set at 2, and the number of estimators is set at 354.

#### Support vector machine

Three different kernels “Linear”, “Poly” and “RBF” apply for SVM models to generates three SVM models. Since SVM models are expected to compare the performance with other main models rather than achieve the best result, SVM models used the default value of parameters in this study.

### Result

#### Experiment result

**Fig. 5:**
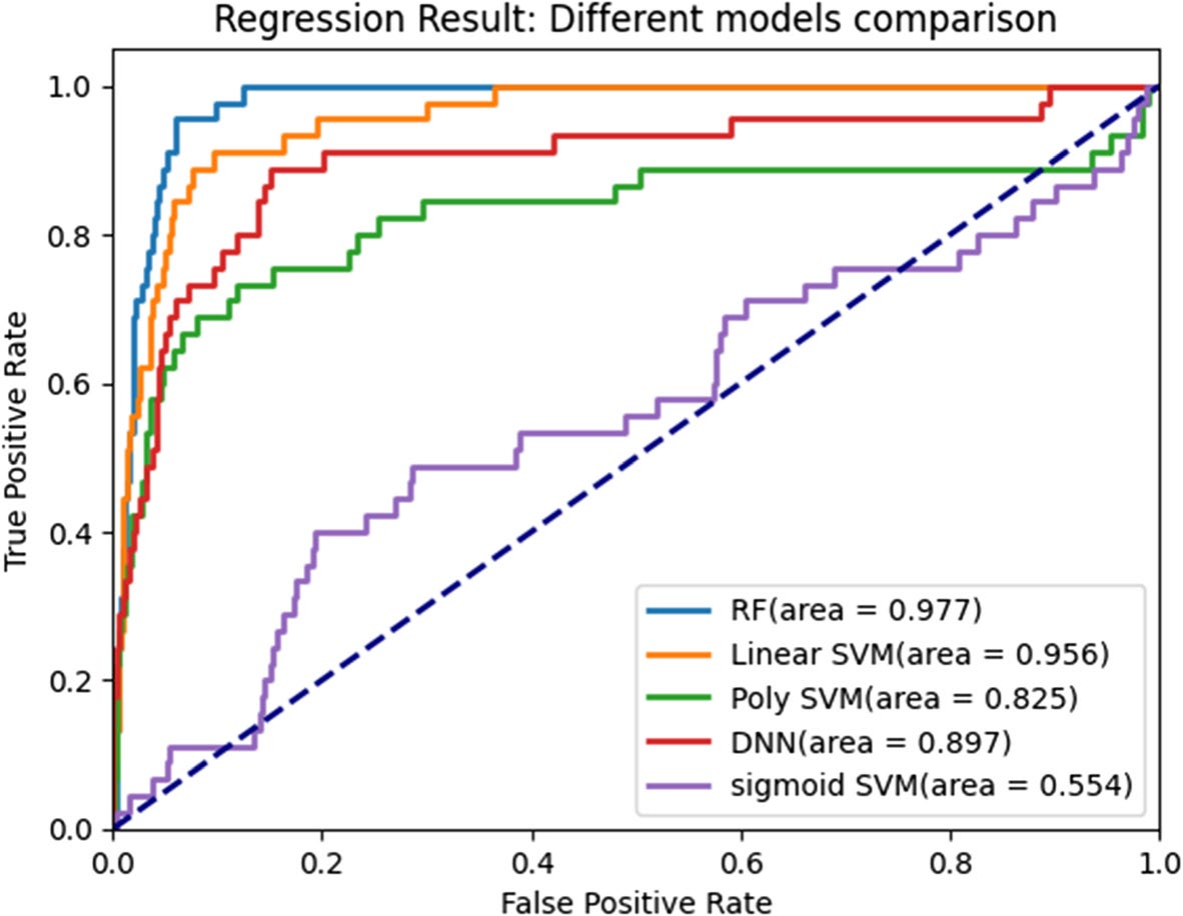
Comparison of ROC curves for all tested models

**Fig. 6:**
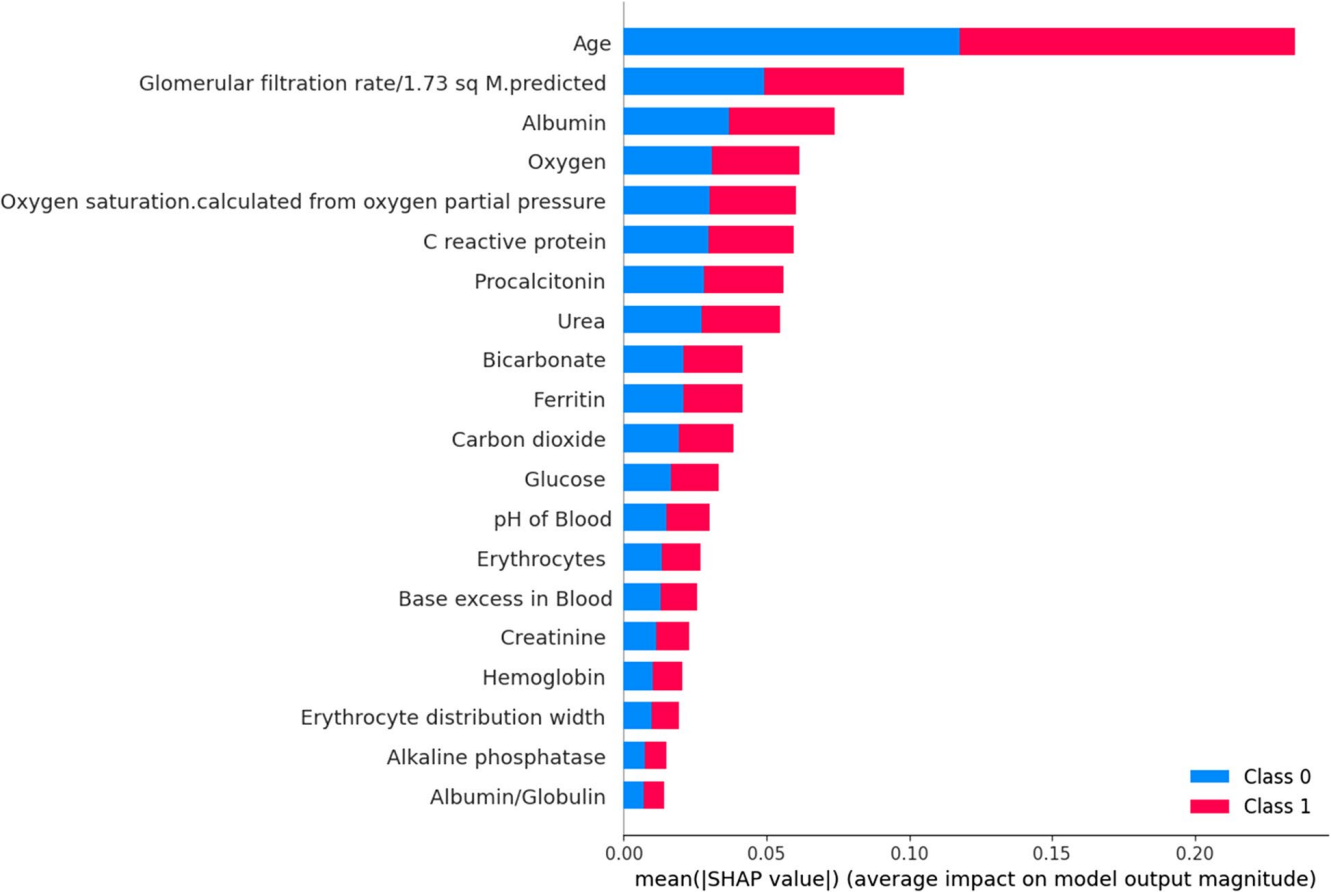
Top 20 immunological and metabolic biomarkers

A performance representation of each of the machine algorithms used in our study under 5-folds cross validation are presented in Fig. 5. Comparison of the models revealed that the random forest model outperformed the others, with an AUC of 0.98 and a 95% CI of 0.89-0.98 for the prospective test set (Figs. 4 and 5). Overall, the significance of our work is multiple folds. For the general purpose, this study provides binary classification result using logistic regression model in the training set and G-mean was used to calculate the optimized threshold. After converting to binary classification, a sensitivity of 0.93 (95% CI: 0.92-0.94), a specificity of 0.93(95% CI: 0.92-0.94), a positive predictive value of 0.28(95% CI: 0.26-0.30) and a negative predictive value of 0.99(95% CI: 0.98-1.00) (Fig. 6). Statistical analysis was per- formed to compare the trained machine learning models using data from our cohort of inpatients from Hong Kong’s public hospitals between January 1, and September 30, 2020, 5,059 were diagnosed with COVID-19 using RT-PCR (n= 5,059) (median age = 46 years; 49.3% male) and 532,427 patients were controls.

The importance of each feature in the data set was calculated using the feature importance package on CityU High performance Computing (HPC). The calculated values are shown in Table 2, and the results obtained with each algorithm when using different numbers of patient records are shown in Table 3.

**Table 2:** Feature importance results and values

Description (Feature name)	Feature value
Age	0.11057
Urea [Moles/volume] in serum or plasma	0.0499
Procalcitonin [Mass/volume] in serum or plasma	0.0390
Albumin/Globulin [Mass ratio] in serum or plasma	0.0357
Magnesium [Moles/volume] in serum or plasma	0.0314
Base excess in blood	0.0298
Creatinine [Moles/volume] in serum or plasma	0.0289
Glomerular filtration rate/1.73 m^2^*predicted*[*VolumeRate*/*Area*]*inserumorplasma* by Creatinine-based formula (CKD-EPI)	0.02623
Calcium [Moles/volume] in serum or plasma	0.0252
Erythrocytes [/volume] in blood	0.02480
Carbon dioxide [Partial pressure] in blood	0.02071
Albumin [Mass/volume] in serum or plasma	0.01949
Lymphocytes/100 leukocytes in blood	0.01921
Lactate dehydrogenase [Enzymatic activity/volume] in serum or plasma	0.0179
Ferritin [Mass/volume] in serum or plasma	0.01778
Creatine kinase [Enzymatic activity/volume] in serum or plasma	0.01737
PH of blood	0.01696
C reactive protein [Mass/volume] in serum or plasma	0.01472

**Table 3:** Comparison of the models performance with different data size

Number of patients	SVM-linear	SVM-Poly	SVM-RBF	SVM-sigmoid	Random Forest	DNN
1,000	0.87	0.74	0.86	0.72	0.91	0.80
2,000	0.89	0.80	0.89	0.50	0.92	0.91
3,000	0.91	0.77	0.87	0.45	0.94	0.92
4,000	0.92	0.77	0.89	0.66	0.98	0.95

**Fig. 7:**
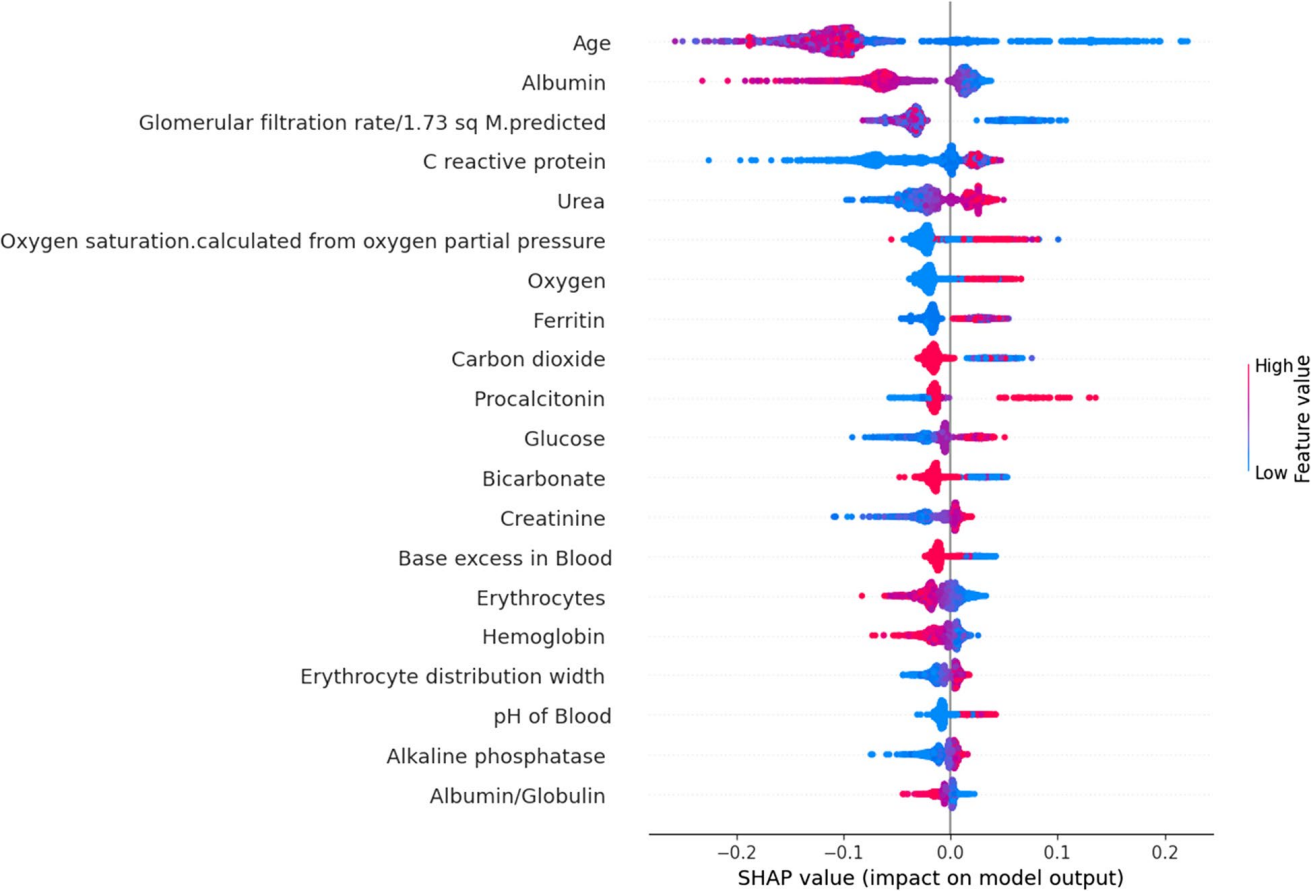
Top 20 immunological and metabolic biomarkers

The top 20 most important immunological and metabolic biomarkers included in the model are ranked in Figs. 6 and 7 and Table 2.

Figures 6 and 7 depict SHapley Additive exPlanations (SHAP) beeswarm plots of the SHAP values for the most important immunological and metabolic biomarkers.

The biomarkers are arranged along each vertical axis by their mean absolute SHAP values. The position of each point on the horizontal axis shows the impact of that feature on the classifier’s ability to predict the outcome of a given COVID-19 patient.

**Fig. 8:**
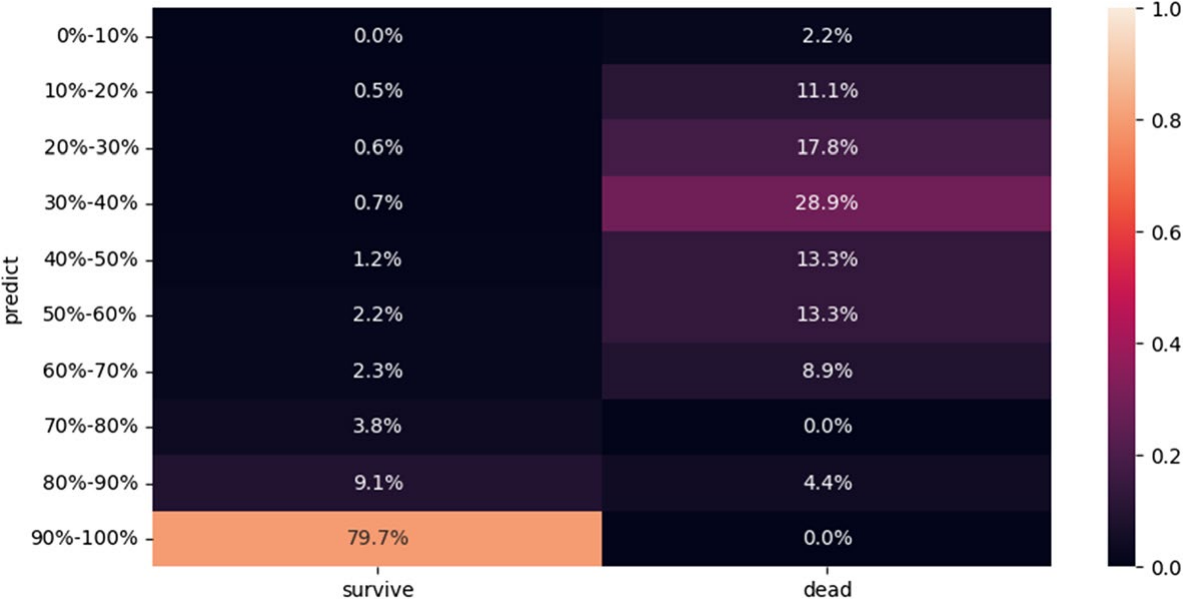
Sample prediction of the risk of mortality

Finally, a sample prediction of the risk of mortality is given using the model that helps in predicting the risk of mortality, as shown in Fig. 8.

#### Validation result

To validate the model, data from a cohort of 131 COVID-19 patients including 124 (who had recovered), six (who were deceased), and one whose situation was unknown, were obtained from the Kwong Wah Hospital. To avoid potential overlap of the data between training, testing and validation sets, patients with a confirmed COVID-19 infection before September 30,2020, were excluded from this validation data set.

Finally, 77 patients were included in the validation dataset, among whom 73 patients had recovered, and 4 were deceased.

**Fig. 9:**
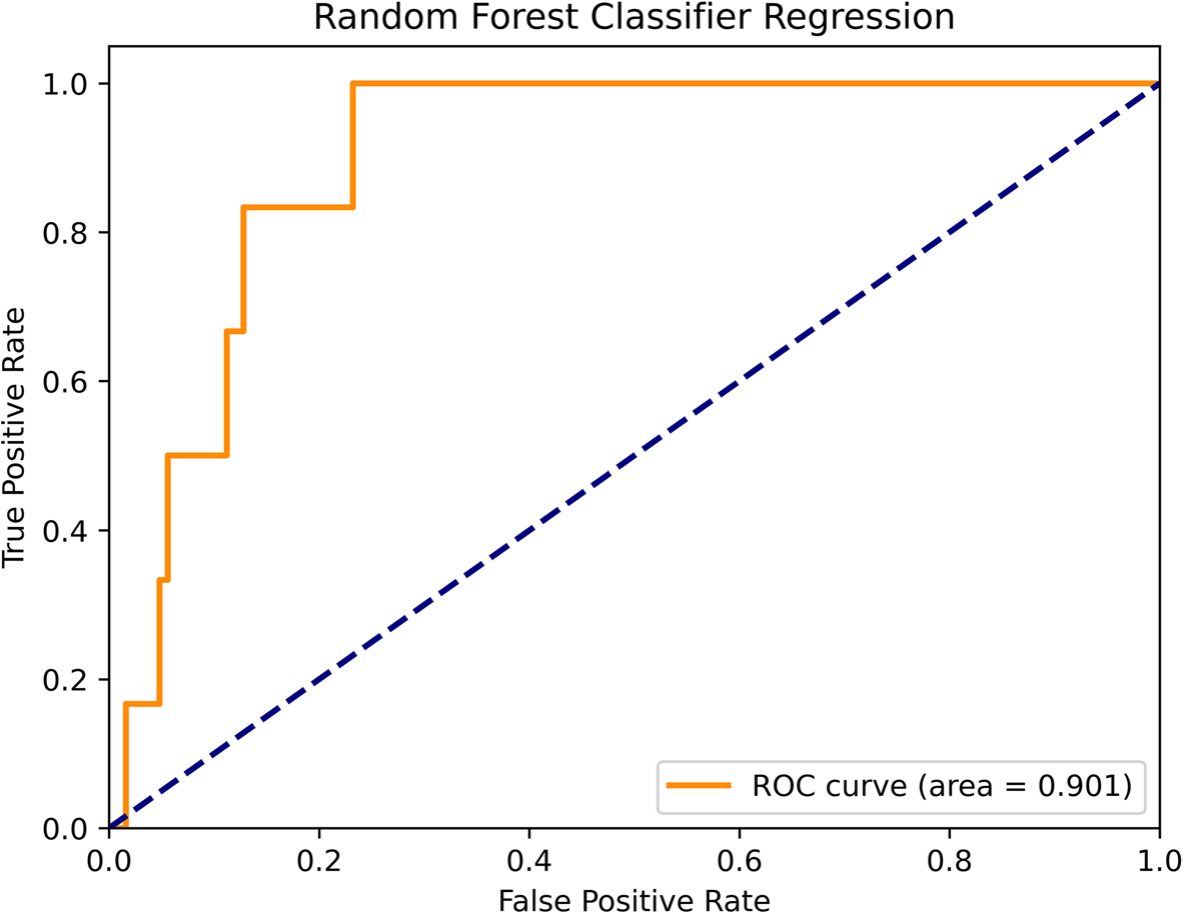
Receiver operating characteristic curve analysis of model validation data

Although only 33 of the 53 input features provided by the hospital was included in the validation set, 18 of the top 20 most important features were included. The results of validation analysis are shown in Fig. 8. An AUC value of 0.90 (0.88-0.92), a sensitivity of 0.67 (0.62-0.69), a specificity of 0.94 (0.92-0.95), a positive predictive value of 0.36 (0.34-0.38) and a negative predictive value 0.98 (0.96-0.98) was obtained for the random forest model (Fig. 9). The model we developed in our study effectively predicted mortality due to COVID-19 based on immunological and metabolic biomarkers in our sample.

## Discussion

In this large study of inpatients from Hong Kong’s Hospital Authority public hospitals between January 1, and September 30, 2020, we identified top 20 immunological and metabolic biomarkers that can accurately predict the risk of mortality from COVID-19 with ROC-AUC of 0.98 (95% CI 0.96-0.98). These biomarkers are hematological, coagulation, cardiac, hepatic, renal and inflammatory factors and can be used by physicians to design treatment strategies to prevent COVID-19 patients from developing critical conditions. Optimization of clinical priorities can reduce the burdens currently faced by health care systems by facilitating optimization of the management of healthcare resources during future waves of the COVID-19 pandemic. The AUC value of the model presented in this paper is higher than most of the related findings so far [12, 13, 17–20].

Nowadays, there are some worldwide scoring systems applied for predicting the mortality risk of COVID-19 [21]. Table 4 shows four commonly used scoring systems which are CURB-65 (confusion, uremia, respiratory rat, BP, age 65 years) [22], International Severe Acute Respiratory Infection terization Protocol-Coronavirus Clinical Characterization Consortium (ISARIC-4C) score [23], National Early Warning Score 2 (NEWS2) [24] and quick COVID-19 Severity Index (qCSI) [25]. Comparing the value of AUC of four scoring systems with the RF model in this study, the RF model shows significantly outstanding performance.

**Table 4:** Comparison of performance with worldwide developed scoring systems

Model	AUC
**RF model(This study)**	**0.98(95% CI:0.89 - 0.98)**
CURB-65 [19]	0.81 (95% CI: 0.68 - 0.94)
ISARIC-4C [20]	0.79 (95% CI:0.78 - 0.79)
NEWS2 [26]	0.86(95% CI:0.84 - 0.88)
qCSI [12]	0.81 (95% CI:0.73 - 0.89)

From a set of algorithms which include efficient machine learning approaches such as DNN, RF, SVM models with linear, polynomial, radial basis function and sigmoid kernels, our analytical experiments demonstrated that the random forest model performed better than the other and identified the top 20 most important immunological and metabolic biomarkers in our study population that help to predict COVID-19 mortality.

Focusing on clinical factors, researchers have previously identified several biomarkers (using an ML-based approach) such as multivariable logistic regression model. A study by [27] showed that the value of D-dimer > 2mg/L was associated with mortality among COVID-19 patients. The group has observed a significant correlation between D-dimer levels and disease severity measured by the CT, oxygenation index, and clinical staging. Another group [28], reported lactic dehydrogenase (LDH), lymphocyte, and high-sensitivity C-reactive protein (hs-CRP) were associated with the survival of COVID-19 patients. In the present study, we applied machine learning-based prediction in a cohort of 5,059 patients (median age =46 years, 95% confidence interval (CI): [45,46.1]; 49.3% male) who had tested positive for COVID-19 based on electronic health records. The study also included 532,427 patients as controls and identified both immunological and metabolic biomarkers that help to predict mortality among COVID-19 patients. Identification of both immunological and metabolic biomarkers is very important for mortality prediction of the COVID-19, which is ever mutating and can lead to serious health conditions.

Our result indicated that age, glomerular filtration, albumin, urea, procalcitonin, c-reactive protein, oxygen, bicarbonate, carbon dioxide, ferritin, glucose, erythrocytes, creatinine, lymphocytes, PH of blood and leukocytes are the most important biomarkers identified to predict COVID-19 mortality which are better suited in the effort of optimizing public health resources, targeted community interventions and clinical decision making. Age was also identified as a key predictor of mortality in previous studies [29]. As with the older age, the immunosenescence and/or multiple medical conditions tend to make patients more prone to critical COVID-19 illness [30]. Lymphocytes are among identified immunological and metabolic biomarkers. They are critical components of the immune system and play very important role in host defense and clearing infections. Medical condition due to lower number of lymphocytes in the blood, is a typical feature in COVID-19 patients and may be a key factor in disease mortality [31]. C-reactive protein, carbon dioxide, oxygen and glucose are also among the identified immunological and metabolic biomarkers that have a significant importance for early diagnosis and mortality because of COVID-19 [31].

Creatinine which is also among our identified immunological and metabolic biomarkers is a waste product made by muscles filtered by kidney. High levels of creatinine indicates that kidneys aren’t functioning properly which in turn has a significant role in predicting COVID-19 mortality [31]. In our study, ferritin and albumin are also among the most important immunological and metabolic biomarkers identified for COVID-19 mortality prediction. Ferritin is a blood protein that contain iron leading to anaemia if low blood ferritin level and albumin is a protein made by liver used as storage reservoir of proteins and transporter of amino acids. Low albumin level on presentation in COVID-19 infection is associated with serious outcomes and mortality [32]. Early identification of high-risk COVID-19 patients is very important, as it can speed up the establishment of more responsive health care systems, ensure instant intervention and intensive care. Besides, early recognition of critical patients can help to mitigate the burden on health systems, enabling the health care providers to prioritize the allocation of limited resources during epidemic peaks and optimize decision-making strategy. To the best of our knowledge, no previous research has identified important immunological and metabolic biomarkers to the extent demonstrated in our study. Our findings cover hematological, coagulation, cardiac, hepatic, renal and inflammatory factors.

This research is not without limitations. We relied solely on data reported by the Hong Kong Health Authority, which may contain biases, sole reliance of Hospital Authority data, utilization of small validation cohort and missing information for some of the features used. All these could lead to lower accuracy of our COVID-19 prediction model focused on immunological and metabolic biomarkers. Despite these limitations, we strongly believe that the machine learning assisted prediction of COVID-19 patient outcomes can help to identify those patients at higher risk of death and thus reduce the mortality rate. This study has room for further improvement which is left for future work. For future research integration of machine learning and SIR/SEIR models is suggested to enhance the existing standard COVID-19 epidemiological models in terms of accuracy and longer lead time. Another limitation for further development will be the analysis’s scalability. Since this study selected a list of specified features focused on COVID19 mortality prediction, it may not be easy to apply the selected biomarkers to other diseases. Still, the methodology of this study can be applied to similar investigations of other diseases with some fine-tuning of the analytical pipeline.

Overall, our study reported 20 important immunity and metabolic biomarkers related to COVID-19 mortality that may lead to scientific insights for the development of immunity and metabolic based treatments. By leveraging the electronic health record data from the Hong Kong Hospital Authority, we provide a systematic approach for precise disease monitoring and risk stratification to effectively tailor clinical care for COVID-19 patients. In particular, we recommend physicians closely monitor haematological, coagulation, cardiac, hepatic, renal and inflammatory factors for potential progression to severe conditions among COVID-19 patients.

## Conclusion

In conclusion, we used territory-wide data reported by the Hong Kong Health Authority to develop a model for predicting COVID-19 mortality risk based on immunological and metabolic biomarkers, which is novel.

Our model was developed after a comprehensive review of a big data set and the highest predictive capacity in the literature. It could be used to assign early prioritized COVID-19 treatment to high-risk patients and enable efficient utilization of public healthcare system recurrently severely stretched by the pandemic.

Finally, we strongly believe that our proposed technique can significantly improve healthcare systems’ decision-making processes regarding precise and targeted medical treatments for COVID-19, enabling medical staff across the globe to triage COVID-19 patients and determine these patients’ health and mortality risks effectively and efficiently.

## Supplementary Information

**Additional file 1.** List of features. It is a document containing list of features in original dataset.

**Additional file 2.** Details of data preprocessing. It is a document describing the details of data preprocessing for data tables.

**Additional file 3.** List of input features. It is a document containing list of input features for the ML models.

## Data Availability

The data extracted and analysed for this study are available from the corresponding author on reasonable request.
