# Supplementary material for: Machine learning-based prediction of COVID-19 mortality using immunological and metabolic biomarkers
Source: BMC Digit Health. 2023 Feb 3;1(1):6. doi: 10.1186/s44247-022-00001-0 (PMC9896457; doi:10.1186/s44247-022-00001-0)
Supplement: Supplementary file 1 — Additional file 1. List of features. It is a document containing list of features in original dataset. [file 44247_2022_1_MOESM1_ESM.docx]

**Supplementary**

**Features name of dataset**

Age

sex

Indicator for COVID-19 patient

alcohol

family_hx_dm

family_hx_ht

family_hx_premat_cvd

height

smoking status

weight

T90(Type of ICPC)

K91(Type of ICPC)

T86(Type of ICPC)

R96(Type of ICPC)

K90(Type of ICPC)

R95(Type of ICPC)

T85(Type of ICPC)

L99(Type of ICPC)

K77(Type of ICPC)

T81(Type of ICPC)

K95(Type of ICPC)

P70(Type of ICPC)

R81(Type of ICPC)

S91(Type of ICPC)

T99(Type of ICPC)

L88(Type of ICPC)

K84(Type of ICPC)

K99(Type of ICPC)

K92(Type of ICPC)

T89(Type of ICPC)

R79(Type of ICPC)

D99(Type of ICPC)

N99(Type of ICPC)

R84(Type of ICPC)

K27(Type of ICPC)

K83(Type of ICPC)

K94(Type of ICPC)

K05(Type of ICPC)

K75(Type of ICPC)

A90(Type of ICPC)

K71(Type of ICPC)

R85(Type of ICPC)

R82(Type of ICPC)

K93(Type of ICPC)

K73(Type of ICPC)

K28(Type of ICPC)

N86(Type of ICPC)

K70(Type of ICPC)

B28 (Type of ICPC)

dft_glucose

fasting_glucose

Value of HbA1c

Seasonal Influenza 2019/2020

Pneumococcal (PCV13)

Pneumococcal (23vPPV)

Hepatitis B

Seasonal Influenza unknown brand

dTap (reduced dose)

HBV Booster (Hypo-responder)

Hepatitis B Immunoglobulins

Seasonal Influenza 2018/2019

Human Papilloma Virus (9v HPV)

Alanine aminotransferase [Enzymatic activity/volume] in Serum or Plasma

Albumin [Mass/volume] in Urine

Alpha-1-Fetoprotein [Mass/volume] in Serum or Plasma

Amylase [Enzymatic activity/volume] in Serum or Plasma

Alkaline phosphatase [Enzymatic activity/volume] in Serum or Plasma

Aspartate aminotransferase [Enzymatic activity/volume] in Serum or Plasma

Base excess in Blood

Bicarbonate [Moles/volume] in Blood

Direct bilirubin [Moles/volume] in Serum or Plasma

Bicarbonate [Moles/volume] in Serum

Bilirubin [Moles/volume] in Serum or Plasma

C reactive protein [Mass/volume] in Serum or Plasma

Calcium [Moles/volume] corrected for albumin in Serum or Plasma

Calcium [Moles/volume] in Serum or Plasma

Calcium.ionized [Moles/volume] in Blood

Anisocytosis [Presence] in Blood by Light microscopy

Carboxyhemoglobin/Hemoglobin.total in Blood

Carcinoembryonic Ag [Mass/volume] in Serum or Plasma

Chloride [Moles/volume] in Serum or Plasma

Cholesterol.in LDL [Moles/volume] in Serum or Plasma by Calculated

Creatine kinase [Enzymatic activity/volume] in Serum or Plasma

Carbon dioxide [Moles/volume] in Blood

Carbon dioxide [Partial pressure] in Blood

Complement C3 [Mass/volume] in Serum or Plasma

Creatinine [Moles/volume] in Serum or Plasma

Creatinine [Moles/volume] in Urine

Creatinine [Moles/time] in 24 hour Urine

Deoxyhemoglobin/Hemoglobin.total in Blood

Erythrocyte sedimentation rate

Iron saturation [Molar fraction] in Serum or Plasma

Fasting glucose [Moles/volume] in Serum or Plasma

Fibrin D-dimer FEU [Mass/volume] in Platelet poor plasma by Immunoassay

Fibrinogen [Mass/volume] in Platelet poor plasma by Coagulation assay

Folate [Moles/volume] in Red Blood Cells

Fractional oxyhemoglobin in Blood

Gamma glutamyl transferase [Enzymatic activity/volume] in Serum or Plasma

Folate [Moles/volume] in Serum or Plasma

Hemoglobin [Mass/volume] in Blood

Lactate dehydrogenase [Enzymatic activity/volume] in Serum or Plasma

Lactate dehydrogenase [Enzymatic activity/volume] in Body fluid

Leukocytes [#/volume] in Blood

Methemoglobin/Hemoglobin.total in Blood

Magnesium [Moles/volume] in Serum or Plasma

Sodium [Moles/volume] in Urine

Nucleated Erythrocytes/100 leukocytes [Ratio] in Blood

Oxygen [Partial pressure] in Blood

Oxygen saturation.calculated from oxygen partial pressure [Mass] in Blood

Osmolality of Serum or Plasma

Osmolality of Urine

pH of Blood

Phosphate [Moles/volume] in Serum or Plasma

Platelets [#/volume] in Blood

Potassium [Moles/volume] in Serum or Plasma

Procalcitonin [Mass/volume] in Serum or Plasma

Protein [Mass/volume] in Serum or Plasma

Protein [Mass/volume] in Urine

Glucose [Moles/volume] in Serum or Plasma

Erythrocyte distribution width [Ratio]

Sodium [Moles/volume] in Serum or Plasma

Troponin T.cardiac [Mass/volume] in Serum or Plasma

Triglyceride [Moles/volume] in Serum or Plasma

Urea/Creatinine [Molar ratio] in 24 hour Urine

Urate [Moles/volume] in Serum or Plasma

Urea [Moles/volume] in Serum or Plasma

Platelet mean volume [Entitic volume] in Blood

Protein [Presence] in Urine by Test strip

Erythrocyte mean corpuscular volume [Entitic volume]

Erythrocytes [#/volume] in Blood

Hematocrit [Volume Fraction] of Blood

Microalbumin/Creatinine [Ratio] in Urine

Albumin/Creatinine [Ratio] in Urine

Albumin [Mass/volume] in Serum or Plasma

Globulin [Mass/volume] in Serum by Calculated

Albumin/Globulin [Mass ratio] in Serum or Plasma

Basophils [#/volume] in Blood

Basophils/100 leukocytes in Blood

Cholesterol [Moles/volume] in Serum or Plasma

Cholesterol.in HDL [Moles/volume] in Serum or Plasma

Cholesterol non HDL [Mole/volume] in Serum or Plasma

Cholesterol.total/Cholesterol in HDL [Molar ratio] in Serum or Plasma

5200293//5200701

Eosinophils [#/volume] in Blood

Eosinophils/100 leukocytes in Blood

Iron [Moles/volume] in Serum or Plasma

Iron binding capacity [Moles/volume] in Serum or Plasma

Iron binding capacity.unsaturated [Moles/volume] in Serum or Plasma

Ferritin [Moles/volume] in Serum or Plasma

Glomerular filtration rate/1.73 sq M.predicted by Creatinine-based formula (MDRD)

Glomerular filtration rate/1.73 sq M.predicted [Volume Rate/Area] in Serum or Plasma by Creatinine-based formula (CKD-EPI)

Hemoglobin A1c (glycated HgB)/Hemoglobin.total [Moles] in Blood

Hemoglobin A1c/Hemoglobin.total in Blood by IFCC protocol

Immature granulocytes/100 leukocytes in Blood

Immature granulocytes [#/volume] in Blood

Lymphocytes [#/volume] in Blood

Lymphocytes/100 leukocytes in Blood

Monocytes [#/volume] in Blood

Monocytes/100 leukocytes in Blood

Myelocytes [#/volume] in Blood by Manual count

Myelocytes/100 leukocytes in Blood by Manual count

Erythrocyte mean corpuscular hemoglobin [Entitic mass]

Erythrocyte mean corpuscular hemoglobin concentration [Mass/volume]

Neutrophils [#/volume] in Blood

Neutrophils/100 leukocytes in Blood

Protein/Creatinine [Mass ratio] in Urine

Protein/Creatinine [Ratio] in Urine

Reticulocytes [#/volume] in Red Blood Cells

Reticulocytes/100 erythrocytes in Red Blood Cells
