# Supplementary material for: Machine learning-based prediction of COVID-19 mortality using immunological and metabolic biomarkers
Source: BMC Digit Health. 2023 Feb 3;1(1):6. doi: 10.1186/s44247-022-00001-0 (PMC9896457; doi:10.1186/s44247-022-00001-0)
Supplement: Supplementary file 2 — Additional file 2. Details of data preprocessing. It is a document describing the details of data preprocessing for data tables. [file 44247_2022_1_MOESM2_ESM.docx]

**Details of Data Preprocessing**

Supplementary Table 1 included the outcome variable of COVID-19 mortality updated to 30 September of 2020, the death date (to month), sex, age, and district. The patient ID was used as a key to merge with other tables. There were 5,019 patients confirmed with COVID-19 including 132 died from COVID-19.

Supplementary Table 2 involved alcohol status with patients classified to 4 categories which are ”Drinker”, ”Ex-drinker”, ”Social Drinker”, and ”Non- drinker”.

Supplementary Table 3 shows the 40 classes of International Classification of Primary Care, 2nd Edition (ICPC-2) codes in the original dataset, codes and the corresponding description. 40 different ICPC-2 codes were consolidated to 17 levels according to its chapter (classified by body systems representing the localisation of the problem and/or disease) and use one-hot coding to generate 17 new columns of ICPC-2 codes.

Supplementary Table 4 shows the ICD-9 codes. We kept the first principal diag- nosis code and principal procedure code. If the first principal diagnosis code and principal procedure code were missing, we replaced it by the first of second principal diagnosis code and second procedure code. The ICD-9 codes were categorical having many values which may lead to overfitting problem and complexity for machine learning models.

Therefore, our Supplementary Table 5 classified ICD-9 codes into nine groups. The corresponding ICD-9 codes used grouping to reduce the dimension of ICD-9 codes, we used one-hot coding to generate the nine new columns.

In the “Family health history of diabetes” dataset, “Family history of heart disease” and “Family history of cardiovascular disease” were merged to the main table directly. The data also included the weight and height information with unification of measurement unites. We calculated the body mass index (BMI) to generate a new feature. BMI is defined as follows:

$$BMI =\frac{Weight\left( kg \right)}{{Height}^{2}(meters)}$$

**Supplementary Tables**

**Supplementary Table 1** Target table of COVID-19 study (n= 537496).

| **Data field** | **Data type** | **Description** | **Remarks** |
| --- | --- | --- | --- |
| Patient id | Numeric | Project-specific serial number for each patient | Mapping key |
| Age | Numeric | Year of birth |  |
| Sex | Character | Sex | ”M” - Male ”F” - Female |
| District | Character | District of residence |  |
| Death date | Numeric | Date of death | Format: YYYYMM |
| Covid-19 | Numeric | Indicator for COVID-19 patient | 0 - No / 1 - Yes |

**Supplementary Table 2** Table of alcohol status (n= 182781).

| **Data field** | **Data type** | **Description** | **Remarks** |
| --- | --- | --- | --- |
| patient pssn | Numeric | Project-specific serial number for each patient | Mapping key |
| ref date | Numeric | Date of reference | Format: YYYYMM |
| alcohol | Character | Alcohol status | Type of Alcohol status:  Drinker Ex-drinker  Social Drinker Non Drinker |

**Supplementary Table 3** Table of ICPC-2 codes chapter.

| **ICPC-2 codes chapter** | **Description** |
| --- | --- |
| A | General and Unspecified |
| B | Blood, Blood Forming Organs and Immune Mechanism |
| D | Digestive |
| F | Eye |
| H | Ear |
| K | Cardiovascular |
| L | Musculoskeletal |
| N | Neurological |
| P | Psychological |
| R | Respiratory |
| S | Skin |
| T | Endocrine, Metabolic and Nutritional |
| U | Urological |
| W | Pregnancy, Childbearing, Family Planning |
| X | Female Genital |
| Y | Male Genital |
| Z | Social Problems |

**Supplementary Table 4** Table of ICD-9 codes.

| **Data field** | **Data type** | **Description** | **Remarks** |
| --- | --- | --- | --- |
| patient pssn | Numeric | Project-specific serial number for each patient | Mapping key |
| admission date | Numeric | Date of inpatient  admission | Format: YYYYMM |
| discharge date | Numeric | Date of inpatient  discharge | Format: YYYYMM |
| los | Numeric | Length of stay (in  days) | Discharge date minus  admission date |
| diag cd 01 | Character | ICD-9-CM principal  procedure code | Based on ICD-9-CM  codes specified of the Data Request Form Non-specified codes are indicated as ”XXX” |
| diag cd 02 - diag cd  15 | Character | ICD-9-CM secondary  procedure code | Based on ICD-9-CM  codes specified of the Data Request Form Non-specified codes are indicated as ”XXX” |

**Supplementary Table 5** Table of ICD-9 codes grouping.

| **Group name** | **ICD-9 codes** |
| --- | --- |
| Circulatory | 390–459, 785 |
| Respiratory | 460–519, 786 |
| Digestive | 520–579, 787 |
| Diabetes | 250.xx |
| Injury | 800–999 |
| Musculoskeletal | 710–739 |
| Genitourinary | 580–629, 788 |
| Neoplasms | 140–239 |
|  | 780, 781, 784, 790–799 |
|  | 240–279, without 250 |
|  | 680–709, 782 |
|  | 001–139 |
|  | 290–319 |
| alcohol | E–V |
|  | 280–289 |
|  | 320–359 |
|  | 630–679 |
|  | 360–389 |
|  | 740–759 |
