# Supplementary material for: Machine learning-based prediction of COVID-19 mortality using immunological and metabolic biomarkers
Source: BMC Digit Health. 2023 Feb 3;1(1):6. doi: 10.1186/s44247-022-00001-0 (PMC9896457; doi:10.1186/s44247-022-00001-0)
Supplement: Supplementary file 3 — Additional file 3. List of input features. It is a document containing list of input features for the ML models. [file 44247_2022_1_MOESM3_ESM.docx]

**Supplementary**

**Input Features**

0 Age

1 smoking status

2 Alanine aminotransferase [Enzymatic activity/volume] in Serum or Plasma

3 Alkaline phosphatase [Enzymatic activity/volume] in Serum or Plasma

4 Base excess in Blood

5 Bicarbonate [Moles/volume] in Blood

6 Bilirubin [Moles/volume] in Serum or Plasma

7 C reactive protein [Mass/volume] in Serum or Plasma

8 Calcium [Moles/volume] corrected for albumin in Serum or Plasma

9 Calcium [Moles/volume] in Serum or Plasma

10 Calcium.ionized [Moles/volume] in Blood

11 Creatine kinase [Enzymatic activity/volume] in Serum or Plasma

12 Carbon dioxide [Moles/volume] in Blood

13 Carbon dioxide [Partial pressure] in Blood

14 Complement C3 [Mass/volume] in Serum or Plasma

15 Creatinine [Moles/volume] in Serum or Plasma

16 Fibrin D-dimer FEU [Mass/volume] in Platelet poor plasma by Immunoassay

17 Hemoglobin [Mass/volume] in Blood

18 Lactate dehydrogenase [Enzymatic activity/volume] in Serum or Plasma

19 Leukocytes [#/volume] in Blood

20 Magnesium [Moles/volume] in Serum or Plasma

21 Oxygen [Partial pressure] in Blood

22 Oxygen saturation.calculated from oxygen partial pressure [Mass] in Blood

23 pH of Blood

24 Phosphate [Moles/volume] in Serum or Plasma

25 Platelets [#/volume] in Blood

26 Potassium [Moles/volume] in Serum or Plasma

27 Procalcitonin [Mass/volume] in Serum or Plasma

28 Protein [Mass/volume] in Serum or Plasma

29 Protein [Mass/volume] in Urine

30 Glucose [Moles/volume] in Serum or Plasma

31 Erythrocyte distribution width [Ratio]

32 Sodium [Moles/volume] in Serum or Plasma

33 Urea [Moles/volume] in Serum or Plasma

34 Platelet mean volume [Entitic volume] in Blood

35 Erythrocyte mean corpuscular volume [Entitic volume]

36 Erythrocytes [#/volume] in Blood

37 Albumin [Mass/volume] in Serum or Plasma

38 Globulin [Mass/volume] in Serum by Calculated

39 Albumin/Globulin [Mass ratio] in Serum or Plasma

40 Basophils/100 leukocytes in Blood

41 Eosinophils/100 leukocytes in Blood

42 Iron binding capacity [Moles/volume] in Serum or Plasma

43 Ferritin [Moles/volume] in Serum or Plasma

44 Glomerular filtration rate/1.73 sq M.predicted [Volume Rate/Area] in Serum or Plasma by Creatinine-based formula (CKD-EPI)

45 Lymphocytes/100 leukocytes in Blood

46 Monocytes [#/volume] in Blood

47 Monocytes/100 leukocytes in Blood

48 Erythrocyte mean corpuscular hemoglobin [Entitic mass]

49 Erythrocyte mean corpuscular hemoglobin concentration [Mass/volume]

50 Neutrophils [#/volume] in Blood

51 Neutrophils/100 leukocytes in Blood

52 Protein/Creatinine [Mass ratio] in Urine
